# Annulus Fibrosus Repair via Interpenetration of a Non‐Woven Scaffold Supports Tissue Integration and Prevents Re‐Herniation

**DOI:** 10.1002/jsp2.70045

**Published:** 2025-02-06

**Authors:** Esteban D. Ongini, Mohammed Abdullah, Julie B. Engiles, Brianna S. Orozco, Andrea Moehl, Ana Peredo, Sonal Mahindroo, Rachel Hilliard, Thomas P. Schaer, Robert L. Mauck, Harvey E. Smith, Mazda Farshad, Jess G. Snedeker, Sarah E. Gullbrand

**Affiliations:** ^1^ University Hospital Balgrist Zürich Switzerland; ^2^ Institute for Biomechanics ETH Zürich Zurich Switzerland; ^3^ Department of Orthopaedic Surgery University of Pennsylvania Philadelphia Pennsylvania USA; ^4^ Corporal Michael J. Crescenz VA Medical Center Philadelphia Pennsylvania USA; ^5^ Department of Clinical Studies, New Bolton Center, School of Veterinary Medicine University of Pennsylvania Philadelphia Pennsylvania USA; ^6^ Zurimed Technologies AG Zürich Switzerland; ^7^ St. Bonaventure University St. Bonaventure New York USA

**Keywords:** biomaterials, biomechanics, imaging, pre‐clinical models

## Abstract

**Background:**

Current surgical management of intervertebral disc herniation often fails to adequately address the risk of recurrence, primarily due to the disc's limited regenerative capacity. Regenerative, biomaterial‐based approaches for tissue augmentation, while showing preclinical promise, have consistently failed to meet the extreme mechanical demands of the intervertebral disc, impeding their clinical translation.

**Methods:**

In this study, we introduce a novel annulus repair strategy that employs the mechanical interpenetration of a non‐woven PET scaffold into intervertebral disc tissue to resist reherniation. We investigate the efficacy in preventing herniations under compression using a bovine explant model and validate its performance in a pilot in vivo study in a goat cervical spine injury model. Healing and scaffold integration are assessed over 4 weeks using computed tomography, magnetic resonance imaging, and histopathology.

**Results:**

We demonstrate that this approach effectively prevents mechanically induced herniation. In vivo, the scaffold interpenetration enables biological integration at 4 weeks post‐surgery, with no evidence of scaffold migration or disc degeneration. The scaffold supports matrix deposition and cell infiltration, with no observed endplate pathologies or osteolysis.

**Conclusions:**

These findings highlight a promising combination of biomechanical reliability and favorable histological outcomes, underscoring the potential of this technology for advancing toward human clinical applications.

## Graphical abstract

Current surgical management of intervertebral disc herniation inadequately addresses the risk of a recurrent herniation, in part due to limited regenerative capabilities of the disc. Here we demonstrate that a new strategy for biomaterial‐based augmentation that harnesses mechanical interpenetration of a non‐woven PET scaffold into the intervertebral disc tissue, using ex vivo and in vivo models.

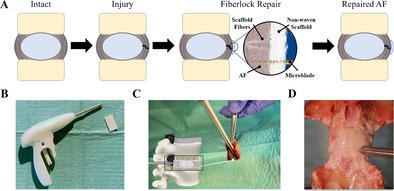

## 1 Introduction

Chronic low back pain is a widespread health condition at the center of disability and decreased quality of life worldwide [1] and is associated with a substantial socioeconomic burden that accounts for 315 billion dollars annually in the United States alone [2]. Intervertebral disc (IVD) herniation and progressive degeneration are believed to be a leading cause of back and neck pain [3, 4, 5]. The main etiology of an IVD herniation is an ongoing degeneration with a loss of hydration in the nucleus pulposus (NP) [6], reducing the hydrostatic pressure acting on the annulus fibrosus (AF) leading to tears [7]. These defects in the AF enable the herniation of NP material, resulting in spinal nerve root compression that induces radiating back pain and motor and sensory deficits [4, 8, 9]. Partial discectomy remains the standard of care for the management of disc herniations, where herniated NP material is removed, effectively alleviating pain and the neurological symptoms [10]. Nevertheless, as discectomy leaves the AF unrepaired, up to 26% of discectomy patients experience poor clinical outcomes [5, 10, 11, 12, 13, 14] due to limited reparative capacities of the AF tissue [15]. When the AF defect is not repaired, the NP can again herniate through the defect [15, 16, 17]. This complication is most prominently observed in patients with large annular defects, where re‐herniation rates can reach 18%–27%, with most of these cases requiring reoperation [18, 19, 20].

Despite considerable research and development efforts devoted to finding clinical treatments that can meaningfully reduce re‐herniation rates, no approach until now has achieved better clinical outcomes. Suture based closure of AF defects are technically challenging and have failed to improve healing, and aggressive removal of NP material, while reducing the risk of re‐herniation appears to come at the cost of accelerated progression of IVD degeneration [5, 21, 22, 23]. Medical device based augmentation has also been tried, although the Barricaid device stands as the sole FDA‐approved product for annular closure to date [24]. This device employs a flexible polyethylene terephthalate (PET) AF occlusion that is anchored in place at the adjacent vertebrae. Despite effectiveness in reducing recurrent herniations [25, 26], a systematic review of the current clinical evidence suggests that the biopolymer occlusion does not integrate well into the disc tissues, and more generally current collective evidence does not convincingly support its clinical use [27]. Altogether, there is thus an unmet clinical need for new surgical techniques and devices for comprehensive and effective management of disc herniations.

The need for improved AF repair has motivated substantial research efforts, particularly toward developing biomaterial scaffolds or sealants that can accelerate healing and prevent re‐herniation of the disc [28, 29, 30, 31, 32, 33]. This includes engineered AF‐like tissue constructs, some of which can match native tissue properties [34, 35, 36]. However, the mechanical demands on the disc and any implanted construct has emerged as a major technical hurdle [24, 37]. AF repairs using injectable biomaterials have shown promise in sealing the AF in large animal models, with some studies suggesting a low incidence of herniation in vivo [38]. However, these same adhesives have exhibited limitations in biomechanical herniation models, where implants have been prone to displacement and extrusion through the AF defect [39]. While many past efforts have successfully increased the failure strengths of the repairs, they have not demonstrated performance significantly superior to discectomy, the standard of care, and do not mitigate risk of re‐herniation [40, 41]. Moreover, as with next‐generation scaffolds for AF repair, some adhesive repairs have shown evidence of endplate osteolysis and inflammation with device placement in large animal in vivo models [32, 33]. These advances, although significant, have thus failed to shift the risk–benefit ratio in favor of translation to clinical practice.

Overcoming these long‐standing mechanical limitations, we describe a repair strategy that interlocks fibers of a non‐woven microfiber scaffold directly into the injured AF. This novel strategy was able to consistently provide sufficient bonding strength to prevent re‐herniation, a major technical achievement. With additional in vivo evidence showing that the interlocked scaffold well supports biological integration to the tissue, we conclude that the approach opens a fresh and promising path for translation of regenerative treatments for IVD herniation.

## 2 Methods

### 2.1 Study Design

The objectives of this study were (i) to prevent IVD re‐herniations after discectomy in an in vitro mechanically induced herniation model by repairing the AF with a PET scaffold using a novel fixation technique (Fiberlock technology), (ii) to demonstrate the feasibility of an annular repair strategy using Fiberlock technology to attach a non‐woven scaffold in a preclinical large animal model, and (iii) to demonstrate the novel repair strategy offers mechanical and biological support for repair at the injury site. We hypothesized the novel fixation technique would reduce the risk of herniation compared to the injured intervertebral disc and would better preserve disc morphology in vivo while allowing for host cell integration.

### 2.2 Materials

PET scaffolds (SpeedPatch PET R, ZuriMED Technologies AG, Zürich, Switzerland) were stretched to 10% strain and then cut to size from a 6 cm by 4 cm piece of material with 1.1 mm thickness. The surgical device used to interlock patch fibers into the AF (FiberLocker Instrument R, ZuriMED Technologies AG, Zürich, Switzerland) was operated at a frequency of 42 Hz.

### 2.3 Motion Segment Preparation for Herniation Risk Assessment

Young adult bovine coccygeal spine segments were procured from a local butcher (Metzgerei Angst, Zürich, Switzerland). Two functional spine units (FSU) of each bovine spine (coccygeal vertebrae 1–2, coccygeal vertebrae 3–4) were dissected to remove irrelevant musculature and ligaments with a scalpel. For each spine segment, the bony endplates were embedded in cylindrical blocks of SCS‐Beracryl D‐28 (Suter Kunsstoffe AG, Fraubrunnen, Switzerland) that were 65 mm in diameter by 35 mm in height with the aid of a potting guide. Specimens were hydrated throughout preparation and testing with a PBS‐soaked cloth. The diameter of the intervertebral discs was measured using a caliper measuring both anterior–posterior diameter and lateral diameter. Specimens were then stored in the freezer (−20°C) until final experimental use with no additional freeze–thaw cycles taking place. Using this methodology, the herniation risk test, conducted under mechanical compression‐only, showed no evidence of loosening.

### 2.4 In Vitro Model for Experimentally Induced Severe Herniation

In vitro assessment of capability to resist re‐herniation was performed using a bovine coccygeal model for experimentally induced severe herniation [30, 39, 41, 42, 43]. To assess the overall capacity to prevent herniations, a worst‐case injury model with a large AF only defect, and a representative injury model simulating the standard of care with a limited nucleotomy were employed. A large 6 × 6 mm cruciate defect, chosen as it corresponds to the clinically identified group of patients at a high risk of re‐herniation [18, 19], was imposed in both injury models with a flat 6 mm wide scalpel blade with a depth stop of 8 mm, followed by NP disruption with a curette. In the simulated discectomy model following AF defect, 200 mg (~20%) of NP tissue was removed from the IVD with a rongeur [39, 40]. Twenty‐eight bovine coccygeal FSUs were randomly allocated, stratifying FSU levels to account for potential effects, into an untreated (injury) group and a group repaired via fiberlocking a PET scaffold (1.6 cm × 1.2 cm) over the injury site (fiberlock) for each injury model with a sample size of seven (*n* = 7/group).

### 2.5 In Vitro Assessment of Capacity to Resist Re‐Herniation

Herniation risk was characterized through a displacement‐controlled (2 mm/min) protocol with stepwise increasing loads to 1.25, 2.5 and 5 kN under 13° flexion to maximize stress at the repair/injury site [40, 44, 45] on a universal material testing machine (Zwick 1456, 20 kN load‐cell, testXpert III; Zwick/Roell, Ulm, Germany). These load steps were chosen to induce pressures at the end of the physiological range (2.3 MPa) in the first step [46] up to supraphysiological pressures above 10 MPa, where the failure of intact discs could occur [39], with specimen cross‐sectional areas ranging between 400 and 600 mm^2^. The following mechanical outcome measures assessing herniation risk were computed from the force‐displacement curves using a custom MATLAB code: ultimate stress required to induce herniations, and herniation frequency after each load step. Ultimate stress to herniation was defined as the load required for the first visible herniation or ultimate load reached during the test (endplate failure or 5 kN) divided by the cross‐sectional area estimated by an ellipsoid with the anterior–posterior diameter and lateral diameter. NP herniation was defined as NP protrusion from the outer radius of the AF, scaffold, or AF‐scaffold interface. Disc diameter at the injury site before and after every loading step was recorded to determine permanent bulging.

### 2.6 Surgery, Tissue Harvesting and MRI

Following IACUC approval at the University of Pennsylvania, 10 large frame goats approximately 3 years of age (9 female, 1 male, obtained from Thomas Morris Inc.) underwent surgery on the cervical intervertebral discs, as described in our prior work [33]. Briefly, intraoperative fluoroscopy was utilized to confirm the location of the C2 through C4 vertebral bodies. The ventral cervical spine was exposed using the anatomical plane between the trachea/esophagus and the carotid sheath via sharp and blunt dissection to expose the anterior annulus of the C2‐3 and C3‐4 intervertebral discs. Each animal received either annular injury only (5 × 2.5 mm cruciate injury with 14G (2.1 mm diameter)), 7 mm deep needle puncture without NP removal (providing an injury with the highest likelihood of herniation) or annular injury and repair via fiberlocking a polyethylene terephthalate (PET) scaffold (1.2 cm x 0.4 cm) over the injury site at either C2‐3 or C3‐4. C4‐5 was used as the healthy control for within‐animal comparison for all outcomes, except for MRI analyses, as detailed below.

Following injury or Fiberlock repair, the incision was closed in layers; the animals were hand‐recovered on a mat under veterinarian supervision and returned to their pen once stable and able to ambulate. There were no complications from the surgical procedure—all animals were ambulatory within 40–60 min from endotracheal extubation and started eating immediately upon return to their stalls. During the study period, animals underwent a daily physical exam by a veterinarian which included assessment of neurologic status. At 4 weeks postoperatively, the animals were euthanized and the cervical spines excised en bloc and stored frozen at −20°C until thawing for MRI scans.

MRI scans of each cervical spine were performed on a 3T scanner (Siemens Magnetom TrioTim). T2‐weighted mid‐sagittal images (5 mm slice thickness, 0.5 mm in plane resolution, TR/TE = 4540/123 ms) were obtained. Pfirrmann grading was performed of each disc from the T2‐weighted images by a blinded observer. A series of images for T2 mapping (6 echoes, TE = 13 ms, 5 mm slice thickness, 0.5 mm in plane resolution) was also obtained. Average T2 maps for each experimental group were generated using a custom MATLAB code, as previously described, to quantify NP T2 relaxation times [47]. Due to cervical level‐to‐level variations in AF and NP T2 and inconsistencies in the availability of mid‐sagittal slices at C4‐C5 in this cohort of animals, C3‐C4 uninjured healthy control disc levels from goat cervical spines (large frame goats, approximately 3 years old) from a separate study [33] (*n* = 6) were used for T2 comparisons.

### 2.7 Ex Vivo Functional Compressive Mechanical Testing

Following MRI, the cervical spines were carefully dissected to isolate vertebra‐disc‐vertebra motion segments. The vertebral bodies were potted in a low melting temperature indium casting alloy (McMaster‐Carr) in custom fixtures and ink spots were placed on the vertebral bone immediately distal and proximal to the disc for optical tracking during testing. An electromechanical testing machine (Instron 5948) was utilized to subject each motion segment to a compression and creep testing protocol in a PBS bath with protease inhibitors at room temperature. The testing protocol consisted of 20 cycles of compression from 0 to −100 N (~0.24 MPa), followed by a 1 s step load to −100 N and a 60‐min hold. The 20th cycle of compression was analyzed using a bi‐linear fit in MATLAB to quantify toe and linear region moduli, as well as transition and maximum compressive strain for each sample [48]. The creep displacement versus time curve was analyzed by fitting to a 5‐parameter viscoelastic constitutive model in MATLAB, as previously described [49].

### 2.8 Micro‐Computed Tomography and Histological Analysis

Following mechanical testing, the samples were fixed in 10% neutral buffered formalin for 1 week at 4°C. After fixation, samples were scanned at an isotropic 10 μm resolution using a Scanco Medical μCT50. The cranial and caudal vertebral endplates (the region between the disc and growth plate) were manually contoured and bone morphometry parameters determined using the Scanco Medical Analyzer software. Following micro‐computed tomography (μCT), samples were decalcified in Formical‐2000 and processed through paraffin. 10 μm thick paraffin mid‐sagittal sections were obtained to capture the area of injury and Fiberlock repair. To visualize cell morphology and pathology, sections were stained with Hematoxylin and Eosin and evaluated by a veterinary pathologist. To visualize collagen fibrils, elastin and bone, a one‐step Mallory‐Heidenhain stain was used on additional sections.

Additional mid‐sagittal sections were used for immunohistochemical analysis of inflammatory cytokines and nerves. Sections underwent deparaffinization and were rehydrated prior to antigen retrieval with Proteinase K for 4 min, followed by blocking of non‐specific binding (Background Buster, Innovex Biosciences Inc.). Sections were incubated overnight at 4°C with pairs of primary antibodies: IL‐6 (Abcam ab66872; 1:200 dilution) and TNFα (Abcam ab6671, 1:200) or PGP 9.5 (Millipore SAB450357, 1:100) and phosphorylated and non‐phosphorylated neurofilament H (1:1 ratio of phosphorylated and non‐phosphorylated, BioLegend 801 701, 1:1000 dilution). All antibodies were diluted using a background reducing antibody dilutant (Agilent Dako S3022). Sections were then incubated in fluorescent secondary antibodies for 60 min at room temperature (Abcam, ab150064 and ab150153, dilution matched to primary antibodies), before cover‐slipping with mounting media containing DAPI and imaging (Zeiss Axio Scan.Z1). Immunofluorescent images were thresholded and expression and localization of targets was assessed via percent fluorescent area and mean fluorescent intensity in within hand drawn regions of interest in the anterior and posterior annulus fibrosus using Fiji.

### 2.9 Statistical Analysis

All quantitative data are presented as mean ± standard deviation. Data analysis was performed using GraphPad Prism (Version 9.2.0. San Diego, CA: GraphPad Software LLC). *p* < 0.05 was set for statistical significance, with *p* < 0.1 set as trends. All data was visually inspected and tested using the Shapiro–Wilk test (alpha = 0.05) for normality. Differences in ultimate stresses were investigated with a one‐way ANOVA followed by pairwise comparisons with Bonferroni‐correction of *p*‐values and Fisher's exact tests were used to examine differences in herniation frequency between the repair and control groups for each injury model in the herniation risk experiment. For the in vivo studies, a one‐way ANOVA with a Tukey's post hoc test was preformed to analyze differences in NP T2 and endplate bone morphometry parameters. A two‐tailed Students' *t*‐test was used to determine differences in normalized disc mechanical properties between injured and Fiberlock repaired discs. Pfirrmann grades and the immunofluorescent analysis (MFI and % Area) were determined via the Shapiro–Wilk test to be not normally distributed, and therefore a Kruskal–Wallis with Dunn's multiple comparisons test was utilized to determine significant differences between groups.

## 3 Results

### 3.1 Fixation of Non‐Woven Scaffolds Through Interpenetration of Fibers Into the AF

**FIGURE 1:**
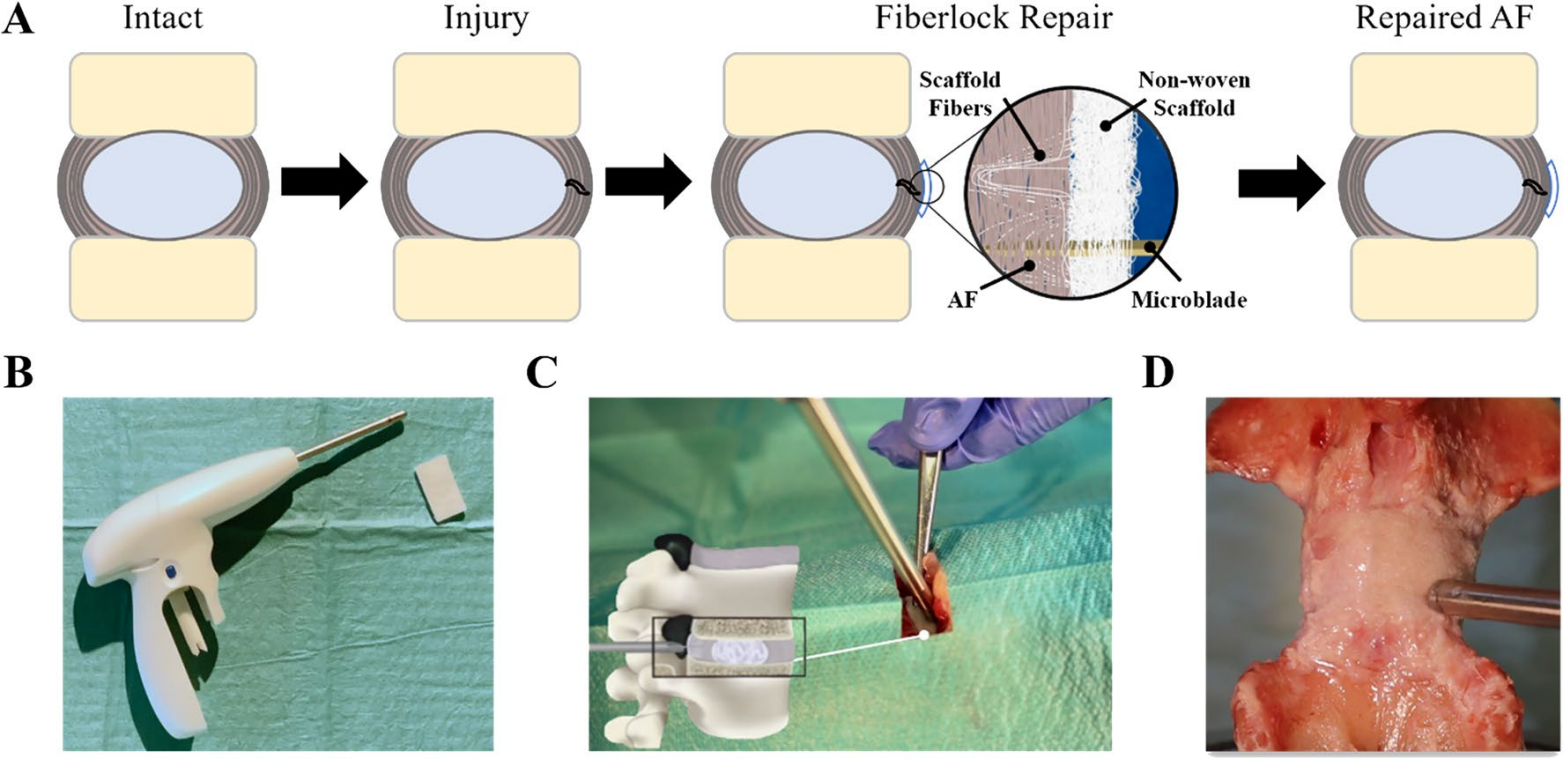
AF repair strategy interlocking non‐woven scaffold fibers into the AF. (A) Schematic illustrations of AF defect repair with Fiberlock technology, showing microblade embedding loose fibers from the PET scaffold into the AF tissue. (B) The surgical application device used to interlock fibers into the AF and PET scaffold. (C, D) Images taken during scaffold implantation on bovine IVDs.

A novel interpenetration technique [50] was successfully adapted for reproducible and robust attachment of a non‐woven PET scaffold to the annulus fibrosus. The configuration of the scaffold was optimized for placement over AF defects as large as 8 mm and designed to act as a mechanical barrier to prevent extrusion of NP tissue through the defect into the extradiscal space under physiological compression. In brief, a micro needle with a 300 μm diameter equipped with barbs (microblade) was used to catch loose fibers within the non‐woven scaffold and pull them into the underlying tissue. In this manner the scaffold was interlocked (“fiberlocked”) with the native AF for mechanical support (Figure 1A,B) enabled by the entangled synthetic and native fibers. The fiberlocking procedure is performed at a high frequency (42 Hz) allowing the attachment of a 1.6 cm × 1.2 cm scaffold in under a minute with a needle penetration of up to 6 mm (Figure 1C,D and Movie S1).

### 3.2 AF Repair With Fiberlocked Scaffold Reduces Risk of Herniation In Vitro

**FIGURE 2:**
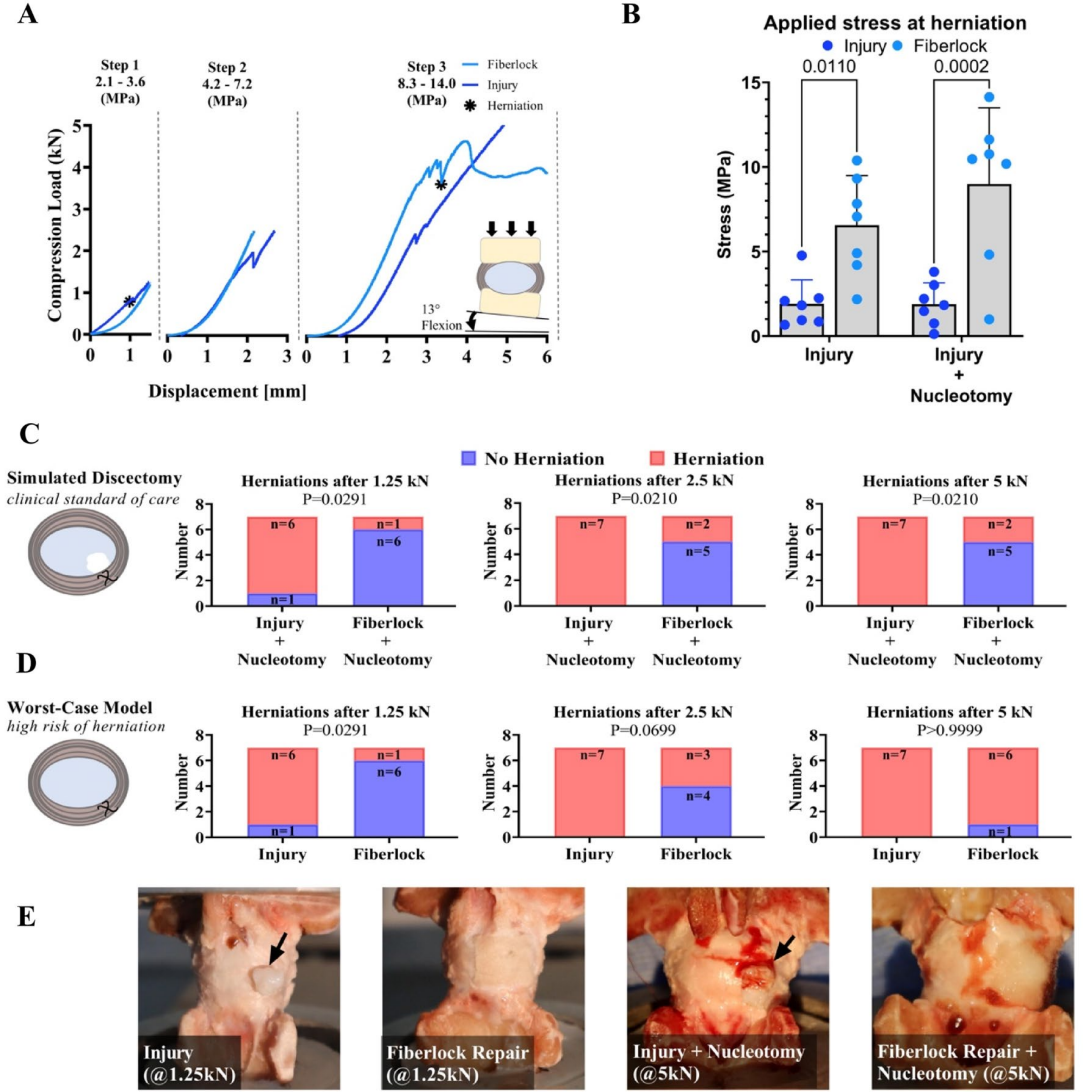
Evaluation of herniation risk following AF repair in a bovine coccygeal model. (A) Representative load–displacement curves of procedure to induce severe herniations for IVDs from an injury and a fiberlocked repair in the worst‐case model. The black asterisk indicates when visual herniation was observed. Intradiscal pressures induced during 1.25, 2.5, and 5.0 kN loading steps are indicated. (B) Stresses required to induce IVD herniation in injury and fiberlocked repairs; bars denote significance (*p* < 0.05). Data are shown as means ± SD (*n* = 7 per group). Significant differences between groups were assessed with a one‐way ANOVA followed by pairwise comparisons with Bonferroni‐correction. (C, D) Herniation frequency following each loading step for the simulated discectomy and in the worst‐case injury model respectively. Fisher's exact tests were used to examine differences in herniation frequency between the repair and control groups. (E) Representative images for injury and fiberlocked repairs following the first load step to peak physiological loads in the worst‐case model and ultimate load for simulated discectomy; black arrows indicate herniated NP.

To quantitatively assess the capacity of the fiberlocked repair to prevent re‐herniation, we characterized the intradiscal stresses required to induce observable herniation. Here employed a representative model of simulated discectomy, reflecting the clinical standard of care, and a worst‐case model, AF without NP removal has been shown to provide the highest likelihood of herniation [39, 43], with a clinically relevant large annular defect through stepwise incremental compressive loading of a bovine functional spine unit (Figure 2A). The bovine coccygeal spine is a standard model to evaluate the mechanical performance of AF repairs, as it has been widely used for investigating the biomechanics of NP/AF implants [39, 40, 41, 43, 51, 52]. Furthermore, herniations are reproducible and bovine discs are large in size with similarities to human lumbar IVDs [53, 54, 55]. The fiberlocked repair provided a clinically relevant increase in the compressive pressure required to induce herniation compared to the unrepaired injury in both the worst‐case (6.55 ± 2.93 vs. 1.91 ± 1.41 MPa, *t* = 3.051, *p* = 0.0110) and in the discectomy model (9.00 ± 4.50 vs. 1.89 ± 1.27 MPa, *t* = 4.669, *p* = 0.0002) (Figure 2B). Following every loading step, the survival of the specimen was observed to quantify the risk of re‐herniation at the upper end of physiological loads and at supraphysiological loads. The fiberlocked repair reduced the risk of herniation compared to simulated discectomy, with a 29% herniation rate in the repair group vs. 100% in controls upon loading to 5 kN (corresponding to induced whole‐disc compression stress of 8.3 to 14 MPa) (*p* = 0.021; Figure 2C). Most of the repaired discs survived the entire test procedure without herniation, with 43% first showing failure of the bony endplates. In the worst‐case injury model, a reduced herniation risk was observed after loading to upper bound of physiological load (1.5 kN) with a 15% herniation rate in the repair group vs. 85% of controls (*p* = 0.029; Figure 2D). In this model of worst‐case defect size, the repair also reduced herniation rates at applied supraphysiological loads, although this was not statistically significant. Notably, specimens with fiberlocked repair herniated at pressures well beyond physiological pressures that would be expected in the intervertebral disc (2.3 MPa), with 86% of herniations occurring above this pressure in both the large defect and discectomy models, whereas only 14% and 29% of the specimens survived these pressures in the respective untreated worst‐case and simulated discectomy model. Herniation exclusively occurred at the defect site (Figure 2E and Movie S2 and S3), and it was observed that in the event of herniation after scaffold‐based repair, a focal region of the patch detached prior to nucleus extrusion.

### 3.3 Fiberlocked PET Scaffolds Closed AF Defects and Mitigated Injury‐Induced Disc Stiffening In Vivo

**FIGURE 3:**
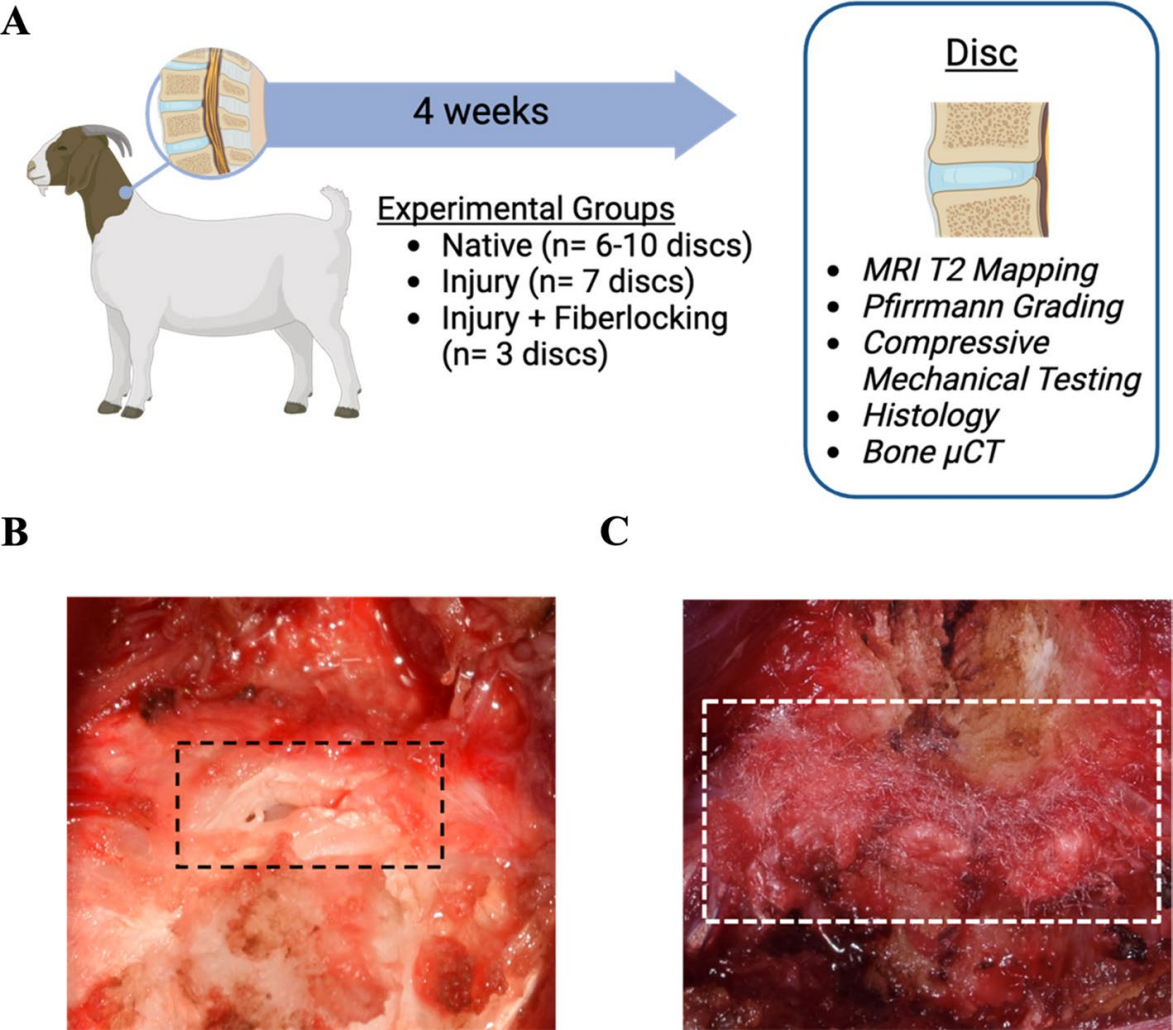
(A) Overview of the in vivo study where goats received either annular injury only or annular injury and repair via fiberlocking with a PET scaffold over the injury site at either C2‐3 or C3‐4. Animals were euthanized at 4 weeks. (B) An image demonstrating the injury (black dashed box) made during surgery, which consisted of a 5 mm × 2.5 mm cruciate laceration (4 mm depth) and a 2.1 mm diameter needle puncture to a depth of 7 mm. (C) Intraoperative image of fiberlocked PET scaffold, with white dashed box indicating location of PET scaffold.

**FIGURE 4:**
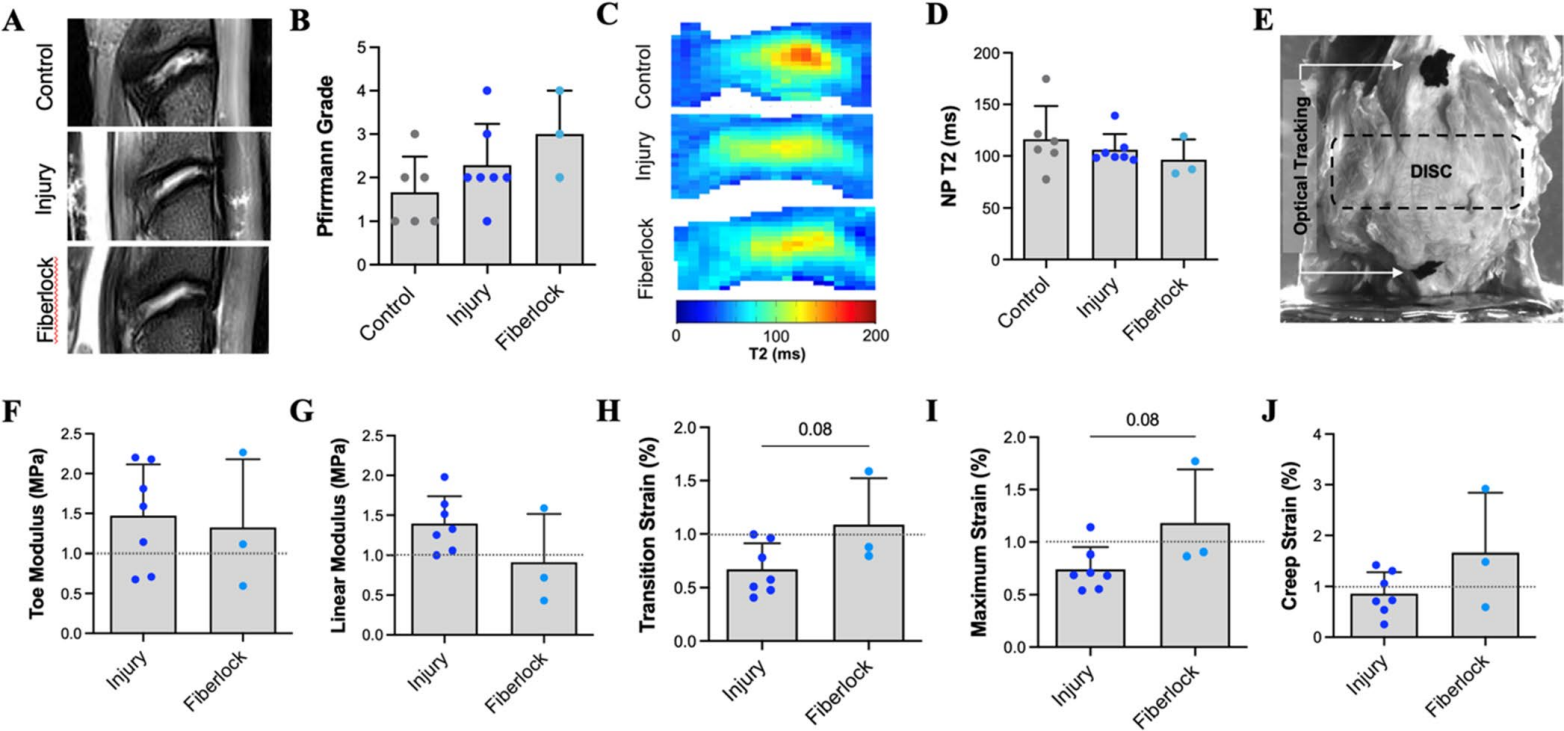
Fiberlocking after AF injury mitigated disc stiffening in vivo. (A) T2 weighted MRIs from each experimental group—control, injury, and fiberlock. (B) Pfirrmann grading for all experimental groups. (C) Pixel‐average T2 MRI maps for each experimental group. (D) Quantification of T2 relaxation times in the NP in each experimental group. (E) Illustration of the compressive mechanical testing, which produced (F) toe region modulus, (G) linear region modulus, (H) transition strain, (I) maximum compressive strain, and (J) creep strain. Data is normalized to the corresponding C4‐C5 healthy control level in each animal. Exact *p*‐values denote trends (*p* < 0.1).

Based on this promising in vitro data, we next assessed the capacity of the fiberlocked PET scaffold to repair an annular injury in vivo, using a large animal study in the goat cervical spine (Figure 3A). The goat cervical spine is a well suited model for mimicking both the human cervical and lumbar spine, due to the disc size (comparable to that of human cervical discs), its semi‐upright nature (in contrast to the horizontal spines of smaller animal models), and comparable biomechanical stresses to the human spine [56, 57, 58]. A total of 10 goats were used in this study, where 7 animals received an injury to the anterior annulus and 3 animals received an annular injury followed by immediate surgical repair by fiberlocking of the PET scaffold (1.2 cm × 0.4 cm) over the injury site (Figure 3B). Unrepaired injury (control intervention) or fiberlock‐based repair (test intervention) was randomized between the C2‐C3 and C3‐C4 levels of the cervical spine in each animal. The annular injury consisted of a partial thickness x‐shaped laceration (2.5 mm × 5 mm, 4 mm deep) of the AF, followed by puncture by a 14G needle through the center of the laceration to a depth of 7 mm without NP removal, creating an injury with the highest likelihood of herniation [33]. None of the animals had any abnormal findings throughout the post‐operative period. Animals were euthanized 4 weeks postoperatively for end‐term analyses. T2‐weighted MRIs demonstrated mild degenerative changes to the discs in both the injury and fiberlock repair group, primarily indicated by diminished distinction between the NP and AF regions (Figure 4A). There were no detectable differences in Pfirrmann grade across all groups (Figure 4B). Quantitative MRI T2 mapping was also used to probe changes in disc hydration or proteoglycan content following injury and repair [59]. Pixel‐average T2 maps of the disc revealed improvements in retention of NP morphology and higher central NP T2 relaxation times with fiberlock repair compared to injured controls, however, when averaged across the NP, no statistically significant differences in T2 relaxation times were observed between groups (Figure 4C,D).

Compressive mechanical testing of isolated vertebral body—disc—vertebral body motion segments (with optical strain tracking) was utilized to determine disc mechanical properties under dynamic physiologic compression and creep loading (Figure 4E). Injury to the AF generally increased overall disc stiffness, with measurably increased linear modulus and reductions in transition and maximal compressive strain when normalized to adjacent healthy control discs from the same animal [33]. Fiberlock repair partially rescued compressive mechanical properties, with similar properties to the adjacent healthy disc. No detectable differences were observed in the toe modulus, linear modulus or creep strain compared to injured discs, however there was a trending increase in transition strain and maximum strain with fiberlock repair compared to injury (*p* = 0.08), indicative of a reduction in disc stiffness (Figure 4G–I).

### 3.4 Fiberlocked PET Scaffolds Support Collagenous Matrix Deposition and Prevents Innervation After AF Injury

**FIGURE 5:**
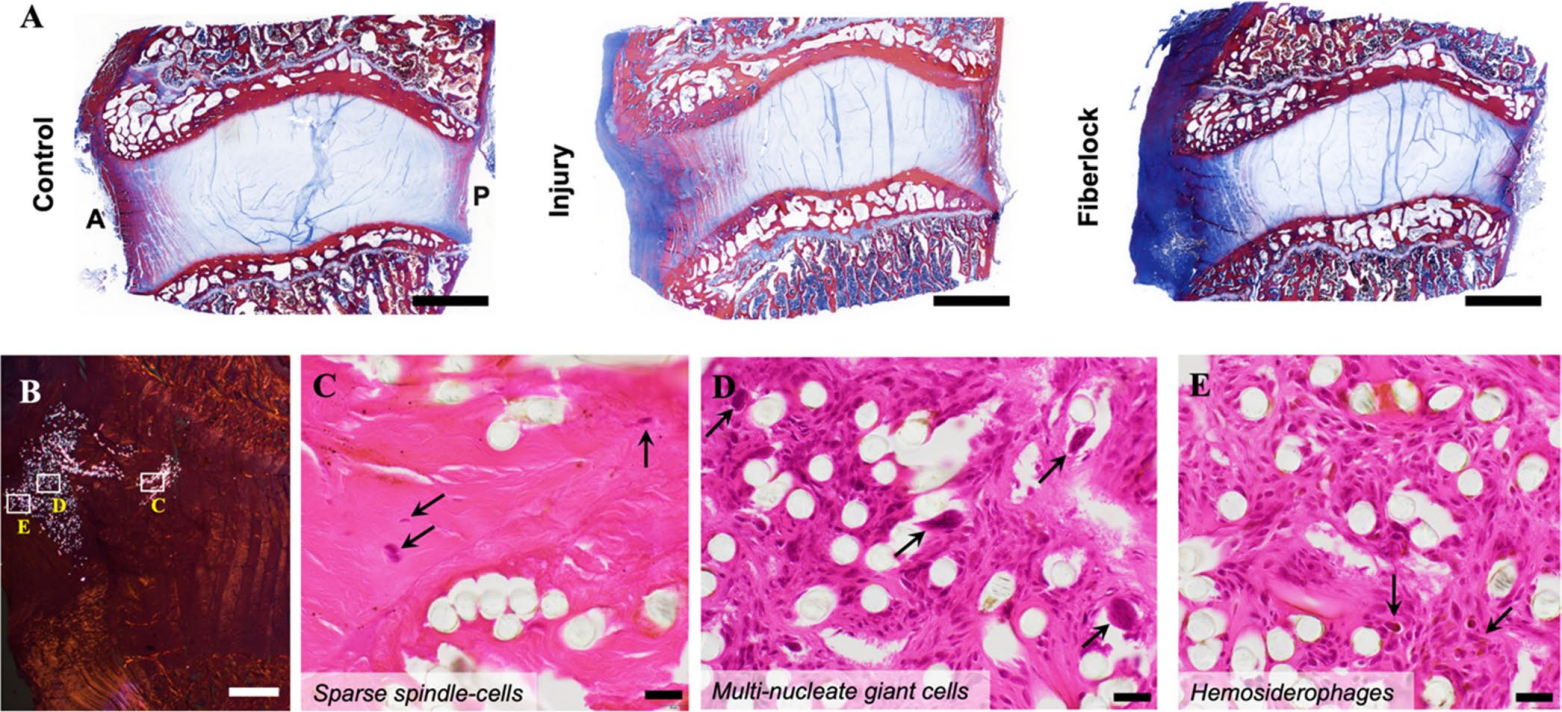
Fiberlocked PET scaffolds support collagenous matrix deposition after AF injury. (A) Representative full disc Mallory Heidenhain‐stained histology (scale = 3 mm) of all experimental groups (A = anterior, P = posterior). (B) Polarized H&E‐stained section including the inner and mid annulus showing integration of the PET scaffold (white signal) and framed locations corresponding to high magnification images (C—E); scale bar = 1 mm. (C) H&E‐stained high magnification image from the junction of the mid‐to‐outer annulus (right‐most white frame in B) showing foci of fiber implant surrounded by paucicellular fibrillar collagenous matrix containing few scattered flattened to plump spindle‐cells (arrows); scale bar = 20 μm. (D) H&E‐stained image of the outer annulus (middle white frame in B) showing moderate cellular infiltrates predominantly comprising mononuclear cells (macrophages, lymphocytes and plasma cells) with scattered multinucleate giant cells (arrows) surrounding individualized implant fibers (clear circles); scale bar = 20 μm. (E) H&E‐stained image of the outer annulus (left‐most white frame in B) showing moderate mononuclear cellular infiltrates and scattered brown pigment‐laden macrophages, compatible with hemosiderophages (arrows), surrounding individualized implant fibers; scale bar = 20 μm.

**FIGURE 6:**
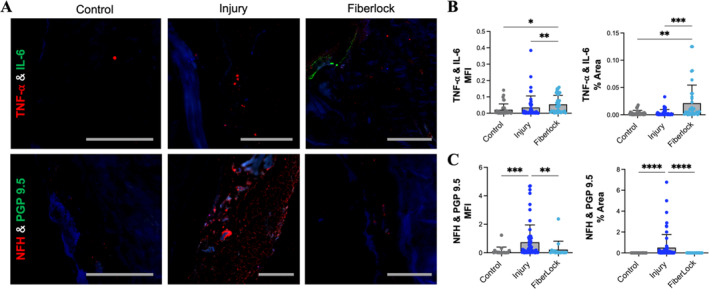
Fiberlocked PET scaffolds prevent innervation after AF injury. (A) Representative immunofluorescence images of the anterior annulus from control, injury, and fiberlock groups, from which the mean fluorescent intensity (MFI) and % area of fluorescence in the anterior and posterior annulus were quantified for (B) TNF‐α (red) and IL‐6 (green) and (C) PGP9.5 (green) and NFH (red) staining. *p < 0.05, **p < 0.01, ***p < 0.001, ****p < 0.0001, scale = 100 μm).

Histological analysis revealed that annular injury resulted in increased fibrous tissue deposition localized within the anterior annulus (Figure 5A). In all animals in the fiberlock intervention group, the PET scaffold was retained at the repair site, and fibers from the scaffold were observed to radially penetrate across several lamellae into the anterior annulus (Figure 5B). Typical of an early healing response to an implanted biomaterial, fibrous tissue infiltration was concurrent with cell infiltrates characteristic of a mild foreign body response and mild prior hemorrhage related to the surgical procedure as indicated by scattered hemosiderophages. This response included activated macrophages, multi‐nucleated giant cells, lymphocytes and plasma cells, visible on Hematoxylin and Eosin staining (Figure 5C–E). Fibrous tissue and inflammatory cell infiltrates were evenly interspersed amongst the implant fibers without evidence of discrete granulomas or neutrophils. Immunohistochemical analysis of inflammatory factors IL‐6 and TNF‐α also demonstrated mild inflammation in the AF of the fiberlock repair group compared to both healthy controls and non‐repaired injured discs (Figure 6A,B). Immunohistochemical analysis of markers of innervation and pain sensation (Neurofilament H and protein gene product 9.5) demonstrated that AF injury was associated with significant increases in innervation in the AF. Notably, AF innervation was significantly reduced with fiberlock repair compared to the injury group, and was maintained at levels similar to that of healthy control discs (Figure 6C).

### 3.5 Fiberlocked PET Scaffold Preserves Vertebral Endplate Morphology

**FIGURE 7:**
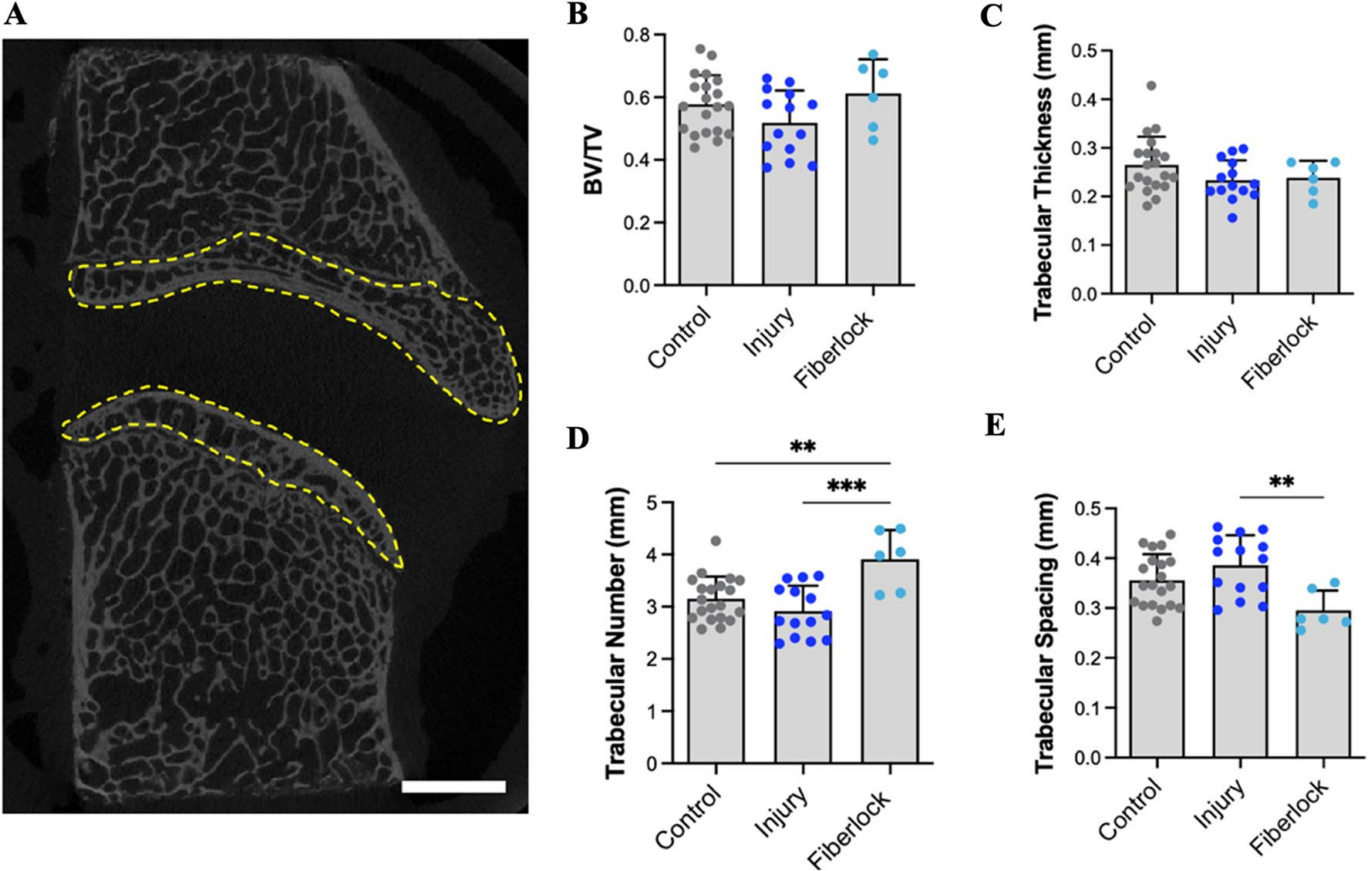
Fiberlocked PET scaffold preserves vertebral endplate morphology. (A) Sagittal μCT slice highlighting the region of the vertebral endplate (yellow dashed lines) that was contoured to quantify bone morphometric parameters for all experimental groups. Scale = 5 mm. (B) μCT quantification of endplate bone volume fraction (BV/TV), (C) trabecular number, (D) trabecular thickness, and (E) trabecular spacing. Cranial and caudal endplates at each level are shown as separate data points. **p < 0.01, ***p < 0.001.

Endplate lesions and osteolysis have been reported to accompany use of the FDA‐approved Barricaid device for annular closure, as well as in pre‐clinical testing of annular repair patches and sealants in large animal models [32, 33, 60]. The vertebral endplate is crucial for spine function and homeostasis, as the vascular channels and marrow spaces within the endplate are the primary nutrient source for the avascular disc [61, 62]. Vertebral endplate morphometry was investigated adjacent to injured and fiberlock repaired discs using μCT scanning and analysis (Figure 7A). No evidence of osteolysis or endplate resorptions (areas of radiolucency on μCT scans) were evident adjacent to fiberlock repaired discs. There were also no detectable differences in endplate bone volume fraction or trabecular thickness across experimental groups (Figure 7B,C). Trabecular number was increased in endplates adjacent to fiberlock repaired discs compared to injured and healthy control discs, accompanied by a reducing in trabecular spacing compared to injured discs (Figure 7D,E).

## 4 Discussion

Partial discectomy and spinal decompression remain the standard surgical treatments for spinal stenosis and disc herniation, especially when disc extrusion compresses nerve roots, causing pain or numbness. However, postsurgical re‐herniation upon mechanical failure of the disc is common, costly and represents a significant unmet clinical need. A treatment that significantly reduces re‐herniation risk compared to the standard of care could lower the necessity for revision surgeries and improve outcomes in the herniated spine. Effective approaches to annular repair have been widely pursued to address this need, however, the extreme mechanical stresses within the IVD have been an obstacle to their advancement.

We employed a novel approach based on mechanical interlocking of fibers from non‐woven scaffold directly with native collagen fibers of the AF tissue. In vitro testing using a widely employed bovine coccygeal IVD model [39, 40, 43], demonstrated that fiberlocking a non‐woven PET patch to an annular defect significantly lowered the risk of re‐herniation with respect to the clinical standard of care, even under extreme biomechanical stress and in the context of large annular defects. Moreover, our data confirm that this mitigation of herniation is effective not only in simulated discectomy but also when the entire NP volume is left intact, representing a biomechanical worst‐case loading scenario closely mirroring a clinical injury condition with a high risk of herniation. Overall, these in vitro data suggest that fiberlock‐based repair offers a clinically meaningful enhancement in mechanical resistance to re‐herniation compared to other current state‐of‐the‐art approaches [30, 39, 40, 41, 42, 44].

Building on these promising in vitro results, we then tested the biological performance of the approach in a large animal goat model of the cervical spine. After 4 weeks, the PET scaffold promoted matrix deposition and integration with the native AF tissue. There was no evidence of scaffold migration or herniation at physiological pressures throughout the study, with fibers from the PET scaffold penetrating several lamellae deep into the AF at 4 weeks, as evidenced from H&E stained histology sections. These findings suggest significant interpenetration of the PET scaffold fibers with the native AF, generating an interface with minimal risk of implant migration. The annular injury model, which did not involve NP tissue removal, imposed a worst‐case loading scenario. All goats in the limited in vivo test series survived without herniation, consistent with our expectations based on the in vitro test results. At the tissue level, no detectable differences in NP hydration were observed across groups, and we observed only statistical trends (*p* < 0.1) in the normalization of disc compressive mechanical properties toward healthy control levels. In the fiberlock intervention group, histological analysis indicated substantial patch integration, with fibrous matrix formation bridging the anterior annulus to the scaffold interlocked across the imposed defect. A mild foreign body response interspersed with the fibers of the non‐absorbable PET implant was observed, characterized by the presence of activated macrophages, multi‐nucleated giant cells and lymphocytes, accompanied by increased expression of inflammatory markers in the anterior annulus. However, endplate architecture was retained, and markers of innervation and pain sensation were reduced compared to untreated injured discs. Taken together, these pilot in vivo results demonstrate initial proof‐of‐concept of fiberlocking for AF repair and the prevention of re‐herniation in a large animal model.

Longer in vivo time points will be needed in future studies to assess the physiologic response to this biomaterial in the annulus and whether the mitigation of neoinnervation within the anterior annulus compared to un‐repaired injury persists. The healthy disc is avascular and aneural, and innervation into the annulus has been observed in herniation and degeneration in both animal models and human tissues, and is posited to contribute to discogenic back pain [63]. Finally, fiberlock repair was not associated with any detectable endplate pathology beyond slight increases in trabecular number compared to injury alone. This is a substantial improvement upon current FDA approved technologies (Barricaid), and next‐generation scaffolds and adhesives for AF repair currently under research investigation—all of which have shown evidence of endplate osteolysis with device placement [32, 33, 60].

An appealing translational feature of the proposed strategy for AF repair employing a PET scaffold is the well characterized and documented biocompatibility of the PET polymer in FDA‐approved devices [64, 65]. Moreover, the attachment process for the scaffold can be achieved through the same surgical accesses as discectomy, can be performed in under a minute in clinically representative defects, and is immediately loadbearing. These present a benefit over injectable biomaterials that cannot achieve similar levels of mechanical support and require several minutes to cure [41, 43] and barrier devices that require complex and time consuming suturing patterns [33], or both invasive disruption of the vertebral endplates and the use of fluoroscopy [66]. While our results are encouraging, they are not without limitations. First, the use of the bovine model for in vitro assessment is limited in its mimicry of typical human cases of relatively young patients with intact facet joints and little to no degeneration [45]. Further, the in vivo testing was done in a small number of animals, and while trends in mechanical differences were observed, the study was underpowered to detect differences in MRI measures of disc composition. MRI measures from fiberlock repaired and injured discs were also only compared to healthy discs from a separate cohort of goats. Given these limited data, the degree to which AF repair alone can protect from degenerative changes and restabilize the IVD following discectomy in vivo has yet to be demonstrated. In future work, a more thorough investigation of the functional mechanical behavior in its six‐axis mechanical loading may also yield more insightful information than the simple compression tests used in the present study.

In summary and in consideration of the study limitations, we demonstrated the feasibility of AF repair using a fiberlocking technique and a non‐woven scaffold. We observed that this approach yielded substantial mechanical and biological support to the injured disc. We conclude that this novel strategy holds potential to reduce the incidence of re‐herniation and revision surgery in discectomy patients and may allow for improved healing of the AF in the herniated spine.

## Supporting information


**Movie S1.** Supporting Information.

**Movie S2.** Supporting Information.

**Movie S3.** Supporting Information.
